# Eavesdropping on Autobiographical Memory: A Naturalistic Observation Study of Older Adults’ Memory Sharing in Daily Conversations

**DOI:** 10.3389/fnhum.2020.00238

**Published:** 2020-06-26

**Authors:** Aubrey A. Wank, Matthias R. Mehl, Jessica R. Andrews-Hanna, Angelina J. Polsinelli, Suzanne Moseley, Elizabeth L. Glisky, Matthew D. Grilli

**Affiliations:** ^1^Human Memory Laboratory, Department of Psychology, University of Arizona, Tucson, AZ, United States; ^2^Naturalistic Observation of Social Interaction Laboratory, Department of Psychology, University of Arizona, Tucson, AZ, United States; ^3^Neuroscience of Emotion and Thought Laboratory, Department of Psychology, University of Arizona, Tucson, AZ, United States; ^4^Evelyn F. McKnight Brain Institute, University of Arizona, Tucson, AZ, United States; ^5^Cognitive Science Program, Department of Philosophy, University of Arizona, Tucson, AZ, United States; ^6^Department of Neurology, Indiana University School of Medicine, Indianapolis, IN, United States; ^7^Minnesota Epilepsy Group, St. Paul, MN, United States; ^8^Aging and Cognition Laboratory, Department of Psychology, University of Arizona, Tucson, AZ, United States; ^9^Department of Neurology, University of Arizona, Tucson, AZ, United States

**Keywords:** episodic specificity, autobiographical memory, episodic memory, semantic memory, cognitive aging, naturalistic observation

## Abstract

The retrieval of autobiographical memories is an integral part of everyday social interactions. Prior laboratory research has revealed that older age is associated with a reduction in the retrieval of autobiographical episodic memories, and the ability to elaborate these memories with episodic details. However, how age-related reductions in episodic specificity unfold in everyday social contexts remains largely unknown. Also, constraints of the laboratory-based approach have limited our understanding of how autobiographical semantic memory is linked to older age. To address these gaps in knowledge, we used a smartphone application known as the Electronically Activated Recorder, or “EAR,” to unobtrusively capture real-world conversations over 4 days. In a sample of 102 cognitively normal older adults, we extracted instances where memories and future thoughts were shared by the participants, and we scored the shared episodic memories and future thoughts for their make-up of episodic and semantic detail. We found that older age was associated with a reduction in real-world sharing of autobiographical episodic and semantic memories. We also found that older age was linked to less episodically and semantically detailed descriptions of autobiographical episodic memories. Frequency and level of detail of shared future thoughts yielded weaker relationships with age, which may be related to the low frequency of future thoughts in general. Similar to laboratory research, there was no correlation between autobiographical episodic detail sharing and a standard episodic memory test. However, in contrast to laboratory studies, episodic detail production while sharing autobiographical episodic memories was weakly related to episodic detail production while describing future events, unrelated to working memory, and not different between men and women. Overall, our findings provide novel evidence of how older age relates to episodic specificity when autobiographical memories are assessed unobtrusively and objectively “in the wild.”

## Introduction

Remembering experiences from our personal history, or autobiographical memories, is thought to aid in the development and maintenance of the self, facilitate social communication, and guide behavior (Pillemer, 1992; Bluck and Alea, 2002; Pasupathi et al., 2002; Bluck, 2003; Bluck et al., 2005). Importantly, the act of recalling and sharing autobiographical memories allows us to communicate a wealth of information with others, from general knowledge to details of a one-time event (Conway and Pleydell-Pearce, 2000), with each form serving distinct purposes (Waters et al., 2014). Such disclosures of personal information in conversation are thought to increase intimacy and empathy within relationships, provide opportunities to teach others, and aid decision making (Alea and Bluck, 2003, 2007). In this sense, autobiographical memory sharing helps us navigate our social interactions.

In prior research, much attention has been given to understanding the relationship between older age and the way autobiographical memories are shared. One reliable finding is that normal cognitive aging is linked to a reduction in the *episodic specificity*, or the frequency of episodic retrieval and/or the vivid elaboration, of autobiographical memories. For instance, prior research has found that, when asked to reflect on the past, older adults retrieve fewer unique life events in comparison to young adults, and in counterbalance recall more events that are extended over time or are repeated events (Piolino et al., 2006; Ros et al., 2009, 2017; Ford et al., 2014). When asked to elaborate these episodic memories, older adults also tend to incorporate fewer episodic details relative to young adults, replacing them with semantic details, or factual knowledge about one’s life story and the broader context of the event (Levine et al., 2002; Addis et al., 2008; Gaesser et al., 2011; Devitt et al., 2017). Critically, some work has further shown that reductions in episodic specificity are evident when comparing young-old to old-old adults (e.g., ages 65–75 vs. +75 or ages 60–69 vs. 70–79), indicating that this effect continues in advanced normal cognitive aging (Piolino et al., 2002; De Beni et al., 2013). The age-related reduction in episodic specificity, broadly reflecting a move away from episodic retrieval, has been interpreted in a few ways. One explanation is that older age is associated with a compensatory shift towards conceptual retrieval that is due to reduced executive resources (Piolino et al., 2010) and stronger coupling of two large-scale brain networks (Turner and Spreng, 2015; Spreng et al., 2018), resulting in a tendency to view things in a semantic light. Alternatively, a natural alteration in narrative style, perhaps related to new perspectives on a lifetime of experiences or changes in language use, could contribute to reduced episodic specificity with age (James et al., 1998; Trunk and Abrams, 2009; Gaesser et al., 2011). Both of these explanations can account for why prior work has commonly found that reduced episodic retrieval is accompanied by increased semantic retrieval. Regardless of the reason(s), these findings and theories suggest that in everyday life, increased age may be linked to less sharing of one’s episodic past.

Despite the vast evidence for age-related reductions in episodic specificity and its potential importance to how memories are shared in everyday social life, this quality of memory retrieval has been largely studied in an analog of the real-world, namely through laboratory interviews (Williams and Broadbent, 1986; Levine et al., 2002; St. Jacques and Levine, 2007; Addis et al., 2008; Barnabe et al., 2012). In one approach, participants generate memories in response to positive and negative cue words, and they are subsequently scored as “episodic” or as more general memories of the past (Williams and Broadbent, 1986). Other laboratory interviews instruct individuals to retrieve episodic memories from a particular time or period and to focus on describing episodic details (Levine et al., 2002). Often, participants are provided cues, such as neutral words (e.g., “tree”), and once a natural ending point is reached, the experimenter may probe for additional information. These reports are then parsed into individual details, with each one scored according to whether it is episodic or “internal” to an event or more generic or “external,” providing a fine-grained, objective picture of recollection (Levine et al., 2002).

Although, there are practical and psychometric benefits to this structured approach, the laboratory interview departs from the contexts encountered naturally in everyday life in several ways, including how autobiographical memories are likely cued and with whom they are commonly shared. Diary studies and other naturalistic thought sampling methods, which require individuals to record events or report on them, have revealed commonalities between laboratory-derived and real-world memories (Levine et al., 2002; Berntsen and Hall, 2004; Schlagman and Kvavilashvili, 2008; D’Argembeau et al., 2011). Yet, by their nature, these methods do not capture in-the-moment, naturalistic outward sharing of autobiographical memories with others, and these studies have not focused on assessing episodic specificity at the level of detail commonly done in the laboratory. Therefore, it remains unclear how age-related alterations in episodic specificity unfold in real-world social contexts.

One way to address this gap in knowledge is to utilize ambulatory assessment technologies that allow for the unobtrusive recording of everyday conversation as it happens (Mehl, 2017). The Electronically Activated Recorder or EAR (Mehl et al., 2001; Mehl, 2017) is one method for such an approach (see Robbins, 2017). The EAR is a mobile smartphone application that periodically samples blocks of ambient sounds, including conversation, from one’s moment-to-moment environment, providing what amounts to an acoustic log of one’s day. Recently, the EAR has been applied to assess autobiographical sharing in natural, everyday social life, revealing outcomes that replicate and go beyond findings from the laboratory. For instance, prior work using the EAR has shown that in everyday social interactions, older adults tend to be past-oriented (Demiray et al., 2018) and share fewer future thoughts than young adults (Brianza and Demiray, 2019). Older adults also appear to share autobiographical memories in their natural social relationships for specific motives (Demiray et al., 2019). The EAR, therefore, presents a unique opportunity to ask whether, in everyday conversation, older age is associated with a reduction in sharing episodic memories and/or episodic detail.

New technologies that unobtrusively capture many memories over time can also be used to further investigate the influence of older age on autobiographical semantic memory. The relationship between older age and autobiographical semantic memory has been difficult to fully appreciate based on laboratory studies for a few reasons, chiefly that participants are typically required to recall a certain number of memories, and the amount of time given to memory sharing is controlled. As such, it remains unclear if older adults, under less constrained conditions, would retrieve autobiographical semantic memories or details at the rates commonly reported in laboratory-based studies. Concerning the real-world, according to both executive coupling (Turner and Spreng, 2015; Spreng et al., 2018) and narrative style accounts (James et al., 1998; Trunk and Abrams, 2009; Gaesser et al., 2011), older age may not alter the tendency to share autobiographical memories but rather reduced episodic retrieval may be counterbalanced by spared or increased sharing of semantic memories and semantic details (Devitt et al., 2017). However, to counter this position, prior research has revealed that broad structural and functional decline in the default network of the brain seems to emerge in older age and continue to unfold with advancing decades of life (Andrews-Hanna et al., 2007, 2019; Fjell et al., 2009), including in regions implicated in semantic memories and social concepts (Lambon Ralph et al., 2012; Andrews-Hanna et al., 2014). According to the integrity hypothesis of default network functioning (Andrews-Hanna et al., 2014, 2019), these findings suggest that while older adults may turn to autobiographical semantic memories and details if retrieval is evoked externally, older age may be connected to a global reduction in the retrieval of autobiographical memories—both episodic and semantic.

To address these questions about the relationship between older age and autobiographical memory sharing in natural social contexts, we conducted a secondary data analysis from a sample of 102 cognitively healthy older adults who, as part of a study on everyday cognition, completed laboratory-based cognitive testing, and for 4 days, wore the EAR (Polsinelli et al., 2020). In the present sample, The EAR recorded for 30 s every 6–18 min except during a 6-h overnight period starting 30 min after participants’ bedtime (*M* = 10:54 pm; range = 9:00 pm–12:30 am). From the EAR recordings, we extracted autobiographical memories and future thoughts being shared by the participants in social situations (Demiray et al., 2018) to understand the sharing frequency of different forms of personal knowledge (i.e., episodic vs. semantic, past and future). We then scored for detailed composition within each instance of autobiographical episodic sharing using the well-established Autobiographical Interview (AI) protocol (Levine et al., 2002).

Based on research previously conducted on autobiographical memory retrieval in the laboratory, we hypothesized that older age would be associated with reduced episodic retrieval in everyday conversation in two ways. First, we expected older age to be linked to a reduction in autobiographical episodic memory sharing. Second, we predicted that older age would be associated with lower generation of episodic details while describing autobiographical episodic memories. Regarding autobiographical semantic memories, according to the executive coupling (Turner and Spreng, 2015; Spreng et al., 2018) and narrative style accounts (James et al., 1998; Trunk and Abrams, 2009; Gaesser et al., 2011) of autobiographical memory and normal cognitive aging, increased age among older adults may be positively associated with the generation of semantic memories and semantic details while describing episodic memories. However, according to the integrity hypothesis of default network functioning (Andrews-Hanna et al., 2014, 2019) increased age among older adults may be associated with a general reduction in autobiographical memory retrieval in social contexts, including semantic memories, which may be revealed through both frequency and semantic elaborateness (i.e., detail generation). Finally, in light of evidence that age-related cognitive differences in autobiographical memory are also reflected in future thinking (Addis et al., 2008; Madore et al., 2014; De Brigard et al., 2016), we expected that findings in the memory domain (e.g., older age linked to reduced episodic memory retrieval) would also be reflected in future thought sharing (e.g., older age associated with less sharing of future episodic thoughts).

To complement these age-related analyses and provide a clearer picture of the degree to which real-world memory sharing mirrors that of laboratory-based findings in older adults, we also examined a few additional features. First, we investigated whether, as shown in laboratory research, episodic specificity of one’s memories is related to how detailed individuals are when they describe future episodic thoughts (Addis et al., 2008; Hill and Emery, 2013). Second, we examined whether two laboratory-based cognitive testing findings are found while sharing memories in the real-world, namely whether greater episodic specificity is associated with better working memory (Addis et al., 2008; Ros et al., 2009, 2017; Piolino et al., 2010), but weakly related to impersonal, laboratory-based measures of episodic learning and memory (Palombo et al., 2015; Grilli et al., 2018a). Third, similar to laboratory findings, we assessed whether episodic specificity is higher in women relative to men when memory sharing is assessed in real-world social contexts (Davis, 1999; MacDonald et al., 2000; Niedźwieńska, 2003; Pillemer et al., 2003; Andreano and Cahill, 2009; Wang et al., 2011; Fuentes and Desrocher, 2013; Grysman and Hudson, 2013; Wang, 2013).

## Materials and Methods

### Participants

The present study utilized data from a previously conducted study on cognitive aging using the EAR technology (Polsinelli et al., 2020). Participants (46 males/56 females) were between the ages of 65 and 90 (*M* = 76.12, *SD* = 6.00) and had, on average, 16.60 years of education (*SD* = 2.32, range = 12–22)^1^. All participants scored ≥25 on the Mini-Mental Status Examination, which indicated that their cognition was within normal limits^2^. Participants provided written informed consent following the Institutional Review Board of the University of Arizona.

### Materials and Procedure

As mentioned, the present study was a secondary data analysis of the study conducted by Polsinelli et al. (2020). Polsinelli and colleagues adhered to the standard EAR protocol (Mehl, 2017), under which the participants are trained on how to use the EAR device before the study period (e.g., how to charge the EAR at night, how to wear the EAR to maximize recording quality). Participants wear the EAR while going about their days, unaware when exactly the device is recording. Through its sampling, the EAR protects privacy (i.e., takes snippets out of their larger conversational context) and enables at-scale empirical studies. Wearing the EAR is minimally bothersome and it has been successfully used, with good acceptance and compliance, in age groups ranging from childhood to old age (3 years to 93 years) and with both healthy and clinical populations. In the present study, compliance with the EAR procedures was high, with only 10 participants experiencing any notable technical difficulties at any point in the study period (i.e., failing to recharge overnight properly). When participants returned their EAR device, they completed a standard EAR evaluation measure (Mehl and Holleran, 2007). On average, participants’ reported low obtrusiveness for themselves (e.g., “To what extent did the EAR impede your daily activities?”; *M* = 1.87, *SD* = 0.64; 1 = “not at all” through 5 = “a great deal”) and bystanders (e.g., “To what extent did the EAR influence the behavior of people around you?,” *M* = 1.94, *SD* = 0.83). Participants wore the EAR for approximately 4 days (a weekend and two weekdays) and gathered, on average, 310 30-s sound file samples (*SD* = 62, range = 91–405). The total number of sound files included only those during which participants were awake and deemed (i.e., coded as) wearing the EAR. The recorded sound files were transcribed as part of the original data analysis plan. For our secondary data analysis, we first determined whether each sound file contained speech by the participant and then whether the files captured any form of autobiographical memories or future thoughts being verbally shared with another person. We then separated this set into four categories, namely autobiographical episodic memories, semantic memories, future episodic thoughts, and future semantic thoughts. Consistent with prior descriptions, we scored autobiographical memories and future thoughts as episodic if they pertained to a specific event occurring at a particular time and place. Memories and future thoughts that lacked such specificity were scored as semantic.

**Figure 1:**
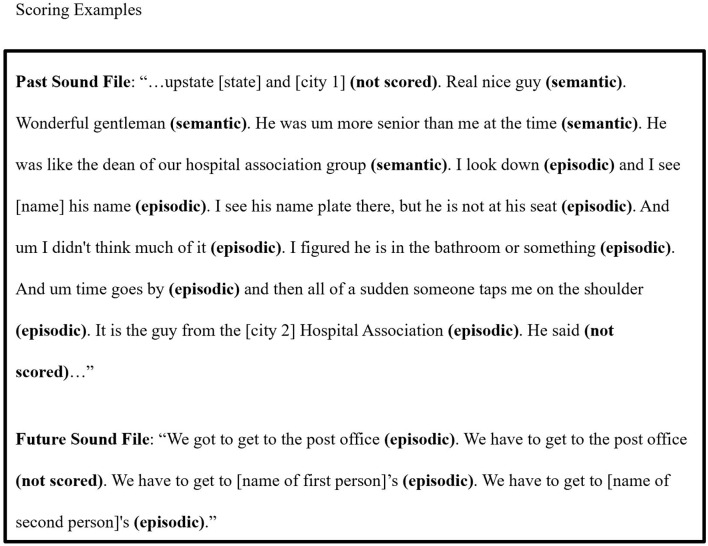
One 30-s sound file that contained a past episodic memory and one 30-s file that contained a future episodic thought. Details at the beginning and end of the past sound file example were not scored because they extended beyond the 30-s recording. In the future sound file example, the second detail was not scored because it was a repetition of the first detail.

We used the AI scoring protocol (Levine et al., 2002) to analyze the content of a subset of these autobiographical sound files. We selected the AI protocol because of its well-validated status as a measure of episodic specificity (Levine et al., 2002; St. Jacques and Levine, 2007; Devitt et al., 2017; Grilli et al., 2018a), its adaptability for scoring future event episodic specificity (Addis et al., 2008; Madore et al., 2014), and neural evidence linking episodic and semantic details to distinct regions/pathways of the default network (Hodgetts et al., 2017; Palombo et al., 2018; Memel et al., 2020). Consistent with the standard AI scoring, all sound files we tagged as containing episodic memories and future episodic thoughts were segmented into individual details and each detail was scored as episodic or semantic. Details were scored as episodic if they described the event content or sequencing, timing, location, perceptual quality, or thoughts or emotions of an event. Details that described facts about the self or world knowledge and details that were personal, non-specific events (e.g., extended or repeated events) were scored as semantic. Each detail was further scored as past or future-oriented based on the temporal nature of the event to which the detail was attached. The original AI protocol includes three additional detail types, namely repetitions, meta-comments, and descriptions of events external to the main event being described in the autobiographical interview. In our application of the AI protocol to EAR sound files, we did not attempt to score these categories. For repetitions and meta-comments, in the absence of insight into the social context, it was often difficult to reliably determine whether someone was absentmindedly thinking aloud, repeating oneself, or sharing information with someone who may not have heard or been present for the initial sharing of that detail. Notably, we did not repeatedly score the same information (e.g., we did not “double score” the re-sharing of the same episodic detail), but we also did not count these details as repetitions. We did not use the external detail category, because we did not assume that a single sound file must capture no more than one episodic memory. Relatedly, although future episodic thoughts might be scored as external details in the laboratory-based application of the AI, we simply scored them as episodic details if attached to episodic memory. To be consistent, we also included future-oriented semantic details attached to episodic memories as semantic details. We want to emphasize that while we slightly departed from the original AI scoring protocol, studies of healthy populations, both young and older, commonly find that semantic details capture the vast majority of “external” contents (Levine et al., 2002; Murphy et al., 2008; Bastin et al., 2013). Figure 1 shows examples of scored sound files.

Two standard cognitive tests that were administered to a subgroup of participants in the original studies were included in this secondary data analysis. One was a test of working memory, measured using the Digit Span Backward subtest raw score from the Wechsler Adult Intelligence Scale, Third Edition (WAIS-III DSB; Wechsler, 1997), and the other was a test of episodic memory, measured with the California Verbal Learning Test, First or Second Edition long delay cued recall raw score (CVLT LDC; Delis et al., 1987, 2000). We selected these two cognitive tests because they have been used in laboratory-based studies that have found relationships between episodic detail generation and working memory (Addis et al., 2008) or have suggested no relationship between episodic detail generation and verbal episodic memory (Grilli et al., 2018a). Only a subset of participants received these tests because, in the original study (Polsinelli et al., 2020), the cognitive battery evolved across two dissertation projects (Polsinelli, 2017; Moseley, 2018), and therefore the entire study sample did not receive the same cognitive tests. Approximately two-thirds of the sample received WAIS-III DSB (*n* = 72, *M* = 7.47, *SD* = 2.26). Twenty-three participants received the first edition of the CVLT LDC, recalling 12.78 words on average (*SD* = 2.56), and 46 received the second edition (*M* = 10.28, *SD* = 2.99). In total, 69 participants had CVLT LDC data from either the first or second edition (*M* = 11.12, *SD* = 3.07)^3^.

### Statistical Procedures

Consistent with established procedures to determine interrater reliability, one primary rater scored all participants and a secondary rater independently scored approximately 20%, or 21 participants (Verfaellie et al., 2014; Grilli et al., 2018b). Cronbach’s alpha was calculated to determine how reliably we could identify the sound files that contained autobiographical memories or future thoughts and the episodic and semantic scoring of the details within the episodic memories and future episodic thoughts.

Two sets of analyses examined the relationship between age and autobiographical memory and future thought sharing. First, to investigate whether age was related to a reduction in the sharing of episodic or semantic memories, we conducted non-parametric (Spearman) partial correlations to examine the relationship between age and sound files that contained autobiographical memories—overall, and both episodic and semantic memories separately (Kim, 2015). Non-parametric analyses were selected given that some data were not normally distributed. The number of sound files that contained any speech from the participant was used as the covariate in these analyses to control for the wide variation of speech production across participants. These analyses were repeated to examine the correlation between age and future thought sharing. Second, to investigate whether age was associated with lower detail generation while describing episodic memories, we conducted Spearman correlations between age and the average number of total details, as well as episodic and semantic details separately, captured per autobiographical episodic memory. A test of correlations from dependent samples with overlapping variables was used to compare the magnitudes of statistically significant partial correlations when appropriate (Steiger, 1980; Diedenhofen and Musch, 2015). This second set of analyses also were conducted on details generated during future episodic thought sharing.

To investigate additional features regarding the quality of social sharing of autobiographical memory, Spearman correlations were conducted to determine if the average frequency of episodic details per future episodic thought description correlated with autobiographical episodic memory description. We also used Spearman correlations to examine the association of the average number of episodic details produced in sound files that contained autobiographical episodic memories with working memory (i.e., WAIS-III DSB) and with a standardized cognitive test of verbal episodic memory (i.e., CVLT LDC). To contextualize our autobiographical memory findings more broadly, we also examined the relationship between age and our laboratory-based measures of working memory and verbal episodic memory. Finally, we conducted an independent samples *t*-test, to investigate gender effects on average episodic detail production (Fox and Weisberg, 2019). All analyses were two-tailed. We did not conduct an *a priori* power analysis and none of the analyses were pre-registered. All analyses and graphs were conducted in or created with RStudio (Wickham, 2007, 2016; Kim, 2015; Auguie, 2017; Fox and Weisberg, 2019; R Core Team, 2019).

## Results

### Interrater Reliability

Good to excellent reliability was achieved for the total number of sound files that contained either an autobiographical memory or future thought, and for episodic and semantic subtypes separately (Cronbach’s alpha range 0.86–0.99). Excellent reliability was also achieved for the total number of details produced across sound files that contained an autobiographical episodic memory or future thought, whether analyzed together or separately (Cronbach’s alpha range 0.91–0.995).

### Sample Included in Autobiographical Memory Analyses

To ensure that individual scores were reliable, we identified four participants who provided fewer than five sound files with an autobiographical memory or autobiographical future thought and an average of 7.50 (*SD* = 3.11) total details. In comparison, the remaining 98 participants produced an average of 27.16 (*SD* = 18.43) sound files with autobiographical memories or autobiographical future thoughts and approximately 84.98 (*SD* = 70.36) total details. Thus, these four participants were excluded based on having too few memories/thoughts and details to likely be reliable. Notably, they were removed before any further analyses of the data. In the remaining sample, the average age was 76.07 (*SD* = 5.94), with 44 men (age: *M* = 75.98, *SD* = 6.49) and 54 women (age: *M* = 76.15, *SD* = 5.51). There was an average of 81.84 sound files (*SD* = 44.04) that captured speech and 23.65 sound files (*SD* = 15.77) that included past autobiographical sharing. Of these sound files, an average of 11.17 (*SD* = 8.95) contained an autobiographical episodic memory, and participants generated an average of 44.24 (*SD* = 42.85) total details for these memories. Also, on average, 2.93 (*SD* = 2.99) focused on future episodic thoughts, with participants generating an average of 6.07 (*SD* = 6.88) total details for these thoughts.

### Relationships Between Age and Autobiographical Sharing

#### Age and the Frequency of Autobiographical Memory and Future Thought Sharing

**Figure 2:**
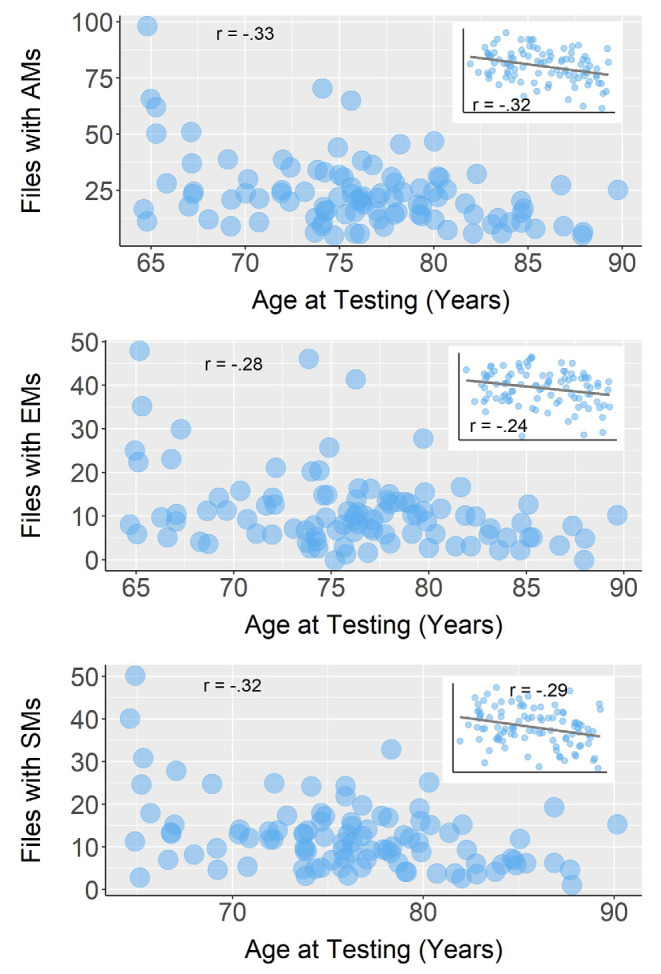
Correlations between age and **(A)** sound files with autobiographical memories (AMs), **(B)** sound files with episodic memories (EMs), and **(C)** sound files with semantic memories (SMs). Raw data and correlation coefficients of the bivariate Spearman correlations are represented in the larger plots. The partial Spearman correlations are shown in the small plots with regression lines. For all three partial correlations, we used a covariate of the number of sound files where participants were engaged in conversation.

When controlling for the amount of conversation captured by the EAR, age was negatively correlated with overall autobiographical memory sharing, *r_s_*_(95)_ = −0.32, *p* = 0.001 (Figure 2). Age was significantly and negatively related to sharing episodic memories, *r_s_*_(95)_ = −0.24, *p* = 0.02, and to sharing semantic memories, *r_s_*_(95)_ = −0.29, *p* = 0.004 (both including amount of conversation as a covariate). A comparison of the magnitude of these two effects revealed that they were not significantly different, *z* = 0.45, *p* = 0.65.

**Figure 3:**
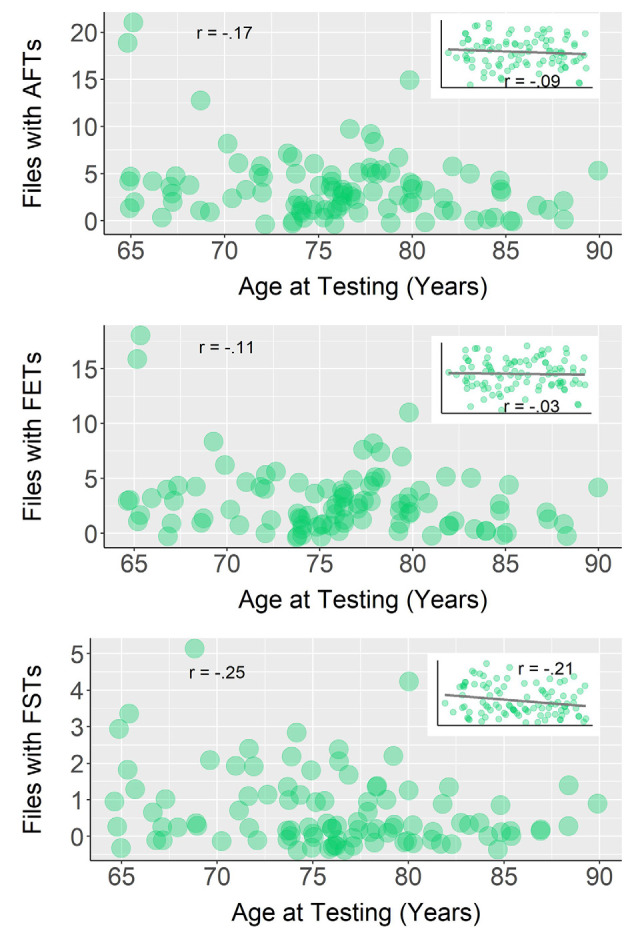
Correlations between age and **(A)** sound files with autobiographical future thoughts (AFTs), **(B)** sound files with future episodic thoughts (FETs), and **(C)** sound files with future semantic thoughts (FSTs). Raw data and correlation coefficients of the bivariate Spearman correlations are represented in the larger plots. The partial Spearman correlations are shown in the small plots with regression lines. For all three partial correlations, we used a covariate of the number of sound files where participants were engaged in conversation.

Alternately, age and autobiographical future thought sharing, covaried for amount of conversation, were not significantly correlated, *r_s_*_(95)_ = −0.09, *p* = 0.38 (Figure 3). However, whereas the correlation between production of future episodic thoughts and age was not significant, *r_s_*_(95)_ = −0.03, *p* = 0.80, the correlation between future semantic thoughts and age was significant and negative, *r_s_*_(95)_ = −0.21, *p* = 0.04 (both including amount of conversation as a covariate).

#### Age and Autobiographical Memory and Future Thought Detail Sharing

**Figure 4:**
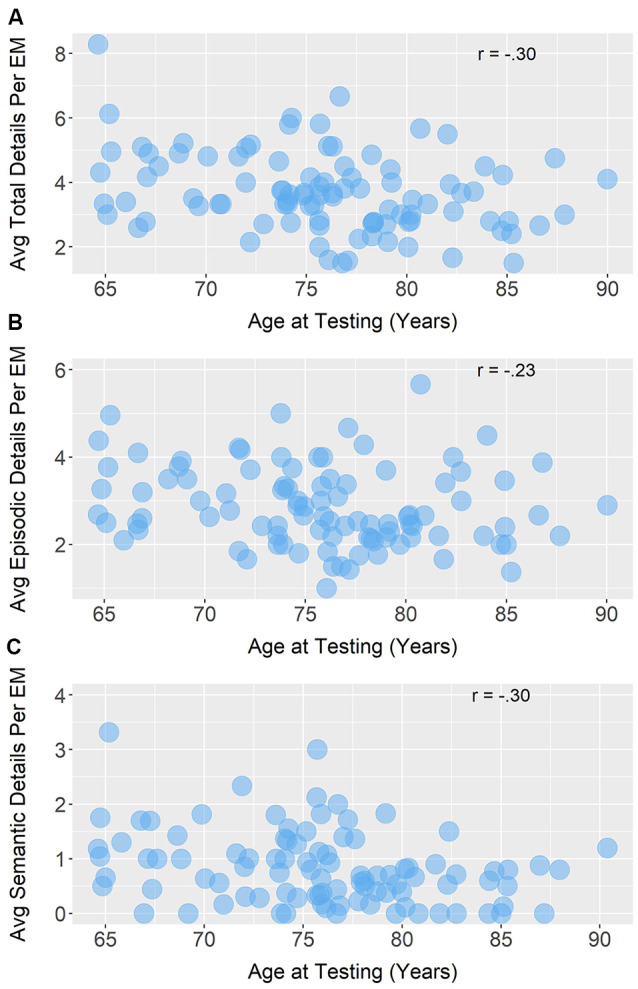
Bivariate Spearman correlations between average (avg) **(A)** total, **(B)** episodic, and **(C)** semantic details produced in sound files containing autobiographical episodic memories (EM) and age.

In regard to past memory detail generation, age was negatively related to the average number of total details produced while describing autobiographical episodic memories, *r_s_*_(94)_ = −0.30, *p* = 0.003 (Figure 4). Age was significantly and negatively related to average episodic, *r_s_*_(94)_ = −0.23, *p* = 0.02, and semantic details, *r_s_*_(94)_ = −0.30, *p* = 0.003, separately. The magnitude of these two relationships did not significantly differ, *z* = 0.57, *p* = 0.57.

**Figure 5:**
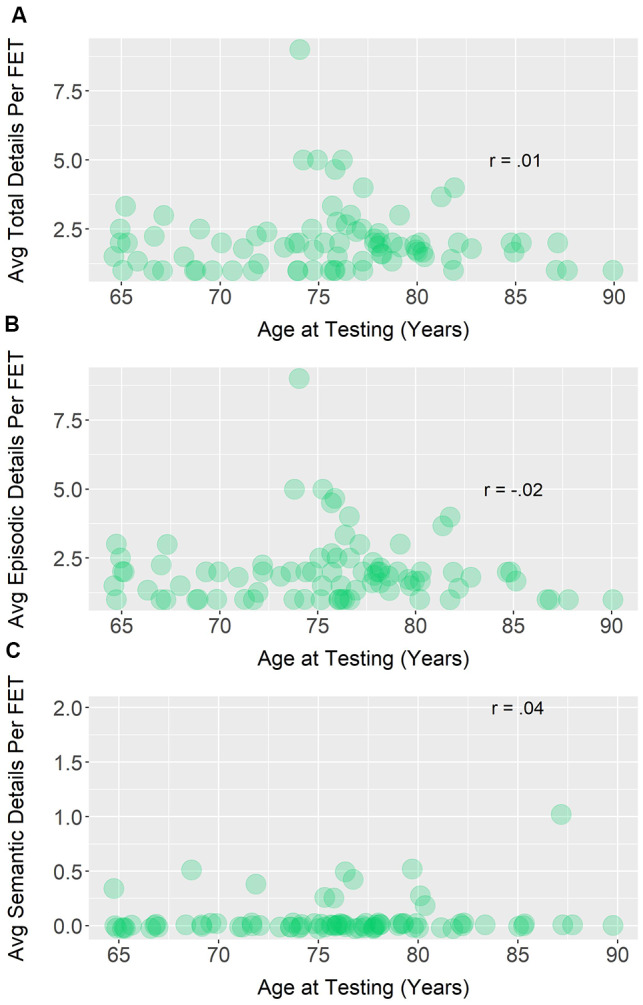
Bivariate Spearman correlations between average (avg) **(A)** total, **(B)** episodic, and **(C)** semantic details produced in sound files containing future episodic thoughts (FET) and age.

For future episodic thought sharing, age and the average total details produced was not significant, *r_s_*_(80)_ = 0.01, *p* = 0.90. Similarly, there was no significant correlation between age and average episodic details, *r_s_*_(80)_ = −0.02, *p* = 0.88, or age and average semantic details, *r_s_*_(80)_ = 0.04, *p* = 0.72. Figure 5 depicts these three relationships.

### Episodic Detail Sharing in Daily Conversation

#### Episodic Detail Generation During Past Episodic Event and Future Episodic Thought Sharing

**Figure 6:**
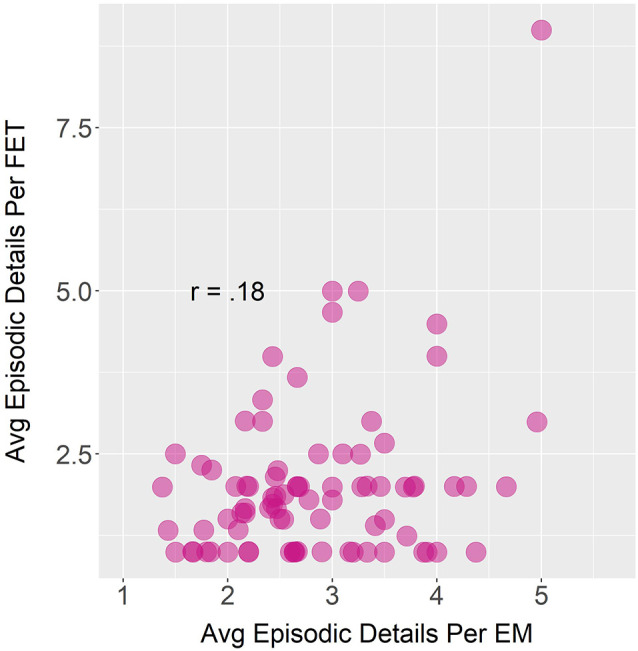
Bivariate Spearman correlation between average (avg) past episodic details and future episodic details generated in sound files with episodic memories (EM) and future episodic thoughts (FET), respectively.

The correlation between the average number of episodic details produced during autobiographical episodic memory sharing and future episodic thought sharing, while positive, was not significant, *r_s_*_(80)_ = 0.18, *p* = 0.10 (Figure 6).

#### Laboratory-Based Cognitive Tests of Working Memory and Episodic Memory

**Figure 7:**
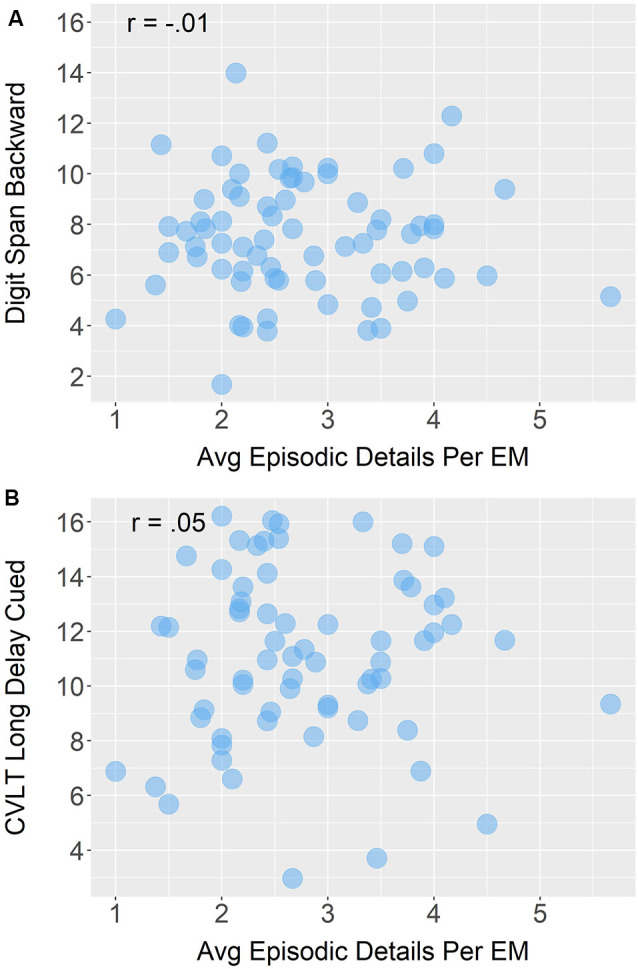
Bivariate Spearman correlations between average (avg) episodic detail generation in sound files that contained episodic memories (EM) and **(A)** measures of working memory (i.e., Digit Span Backward raw score) and **(B)** episodic memory (i.e., California Verbal Learning Test [CVLT] long delay cued recall raw score). The jitter of data points was used to ensure minimal overlap, which pushed some CVLT scores of 16 (maximum score) above that value.

Contrary to laboratory-based findings, our measure of working memory, DSB, was not significantly correlated with the average number of episodic details produced when participants shared autobiographical episodic memories in daily conversation, *r_s_*_(68)_ = −0.01, *p* = 0.96. However, consistent with laboratory-based findings, the association between a laboratory measure of episodic memory (i.e., CVLT LDC) and episodic detail provided while sharing autobiographical episodic memories was also not significant, *r_s_*_(65)_ = 0.05, *p* = 0.66. See Figure 7 for both correlations. Interestingly, age was not significantly correlated with our laboratory-based cognitive measure of working memory, *r_s_*(68) = −0.08, *p* = 0.49, or verbal episodic memory, *r_s_*(65) = −0.12, *p* = 0.32.

#### Gender Comparison of Episodic Detail Generation

**Figure 8:**
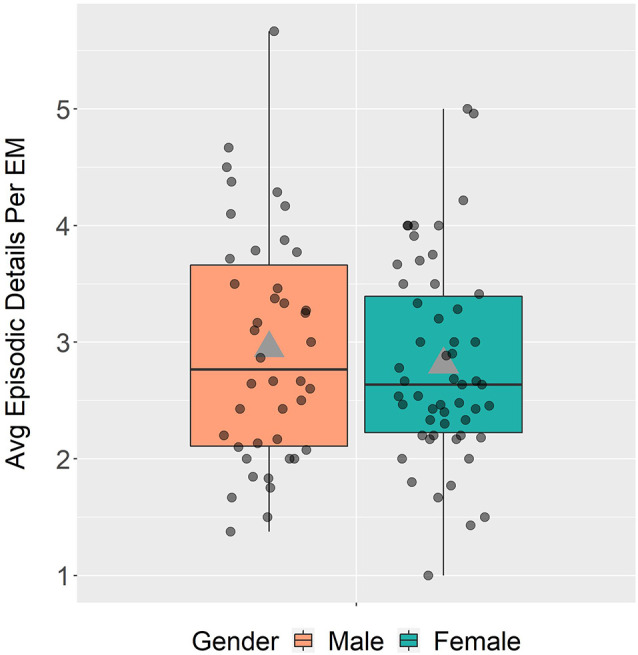
Average (avg) episodic details per episodic memory (EM) in males and females.

Contrary to some laboratory-based findings, men (*M* = 2.95, *SD* = 1.00) and women (*M* = 2.81, *SD* = 0.85) produced comparable averages of the number of episodic details when describing episodic memories, *t*_(94)_ = 0.73, *p* = 0.47, 95% CI (−0.24, 0.51; Figure 8). Co-varying for age did not alter these outcomes, *p* = 0.50.

## Discussion

Individuals share autobiographical memories to juggle a wide range of everyday social situations, including navigating social bonds, teaching others, and solving problems (Pillemer, 1992; Bluck and Alea, 2002; Pasupathi et al., 2002; Bluck, 2003; Bluck et al., 2005). Prior laboratory-based research has extensively studied how the sharing of autobiographical memories appears to change with older age, with one key finding being that there is a robust age-related reduction in episodic memories that is evident in both the frequency at which such memories are retrieved (Piolino et al., 2006; Ros et al., 2009, 2017; Ford et al., 2014) and how much episodic detail is shared to describe unique life events (Levine et al., 2002; Addis et al., 2008; Gaesser et al., 2011; Devitt et al., 2017). The present study utilized the EAR, a method that has recently been applied to capture memories being shared in everyday conversations as they happen (Demiray et al., 2018, 2019; Brianza and Demiray, 2019), to extend the assessment of episodic specificity out of the laboratory and shed light on how older age and episodic specificity are related in the real-world.

By tracking the frequency with which autobiographical memories were generated in social conversations over 4 days, we found that, in an older adult sample, older participants shared fewer autobiographical episodic memories than younger participants. Interestingly, older age also was associated with reduced autobiographical semantic memory sharing in daily conversation. Notably, the magnitude of the effects of age on episodic memory and semantic memory sharing were similar, and not significantly different when directly compared. Together, these findings suggest that older age was linked to a global reduction in the social sharing of autobiographical memories, independent of how often individuals conversed. These results extend our knowledge of the relationship between age and episodic memory by showing that a reduction in the retrieval of episodic memories is not only evident in the laboratory (Piolino et al., 2006; Ros et al., 2009, 2017; Ford et al., 2014), but also during memory sharing in daily conversations. Our findings further suggest that age-related reductions in memory retrieval may not be specific to episodic reflections, at least when autobiographical memory is assessed in daily conversations in the context of increased age among older adults. We interpret these findings as consistent with the integrity hypothesis of default network functioning (Andrews-Hanna et al., 2014, 2019), which predicts that age-related decline in the functionality of the default network should result in a broad reduction in the frequency at which one’s thoughts drift towards the personal past.

Regarding details attached to autobiographical episodic memories, we found a significant negative relationship between age and overall detail generation. Interestingly, older age was not exclusively linked to less episodic detail production, as there was a significant negative relationship between age and semantic detail as well, consistent with the integrity hypothesis of default network functioning (Andrews-Hanna et al., 2014, 2019). The relationship between age and episodic detail did not differ statistically from the magnitude of the age and semantic detail relationship. Thus, similar to our analyses of overall memory sharing, our findings likely suggest that older age has a global, negative effect on memory detail generation while sharing episodic memories. While prior work indicates that reductions in episodic detail may be relevant to social cognitive behavior, including social problem-solving (Vandermorris et al., 2013; Madore and Schacter, 2014) and empathy or intentions to help others (Gaesser, 2013; Gaesser and Schacter, 2014), future research can investigate the broader implications of less semantic detail retrieval in social recollection.

Relative to the autobiographical memory findings, our naturalistic observation of autobiographical future thought-sharing produced weaker relationships with older age. These outcomes are surprising, considering the theoretical and empirical link between remembering and imagining (Schacter and Addis, 2007; Addis et al., 2008; Madore et al., 2014; Addis, 2018). Interestingly, whereas the negative correlation between older age and episodic future thought sharing was not significant, there was a significant, *negative* association between older age and future semantic thought sharing. Therefore, for both remembering and imagining in daily conversation, older age was linked to less outward retrieval of semantic information. On the one hand, these findings are difficult to reconcile with an executive coupling framework (Piolino et al., 2010; Turner and Spreng, 2015; Spreng et al., 2018) or narrative style differences (James et al., 1998; Trunk and Abrams, 2009; Gaesser et al., 2011), and seem more consistent with the integrity hypothesis of default network functioning (Andrews-Hanna et al., 2014, 2019). On the other hand, the low number of future thoughts captured for many participants raises some concerns about the stability of these findings. Similarly, the low frequency of future thought sampling may have compromised the reliability of our estimates of individual differences in details attached to future thoughts, which may explain why we did not find a significant association between age and episodic or semantic detail generation while describing future episodic thoughts. The low frequency of future thought sampling also may be relevant to understanding why we did not find that individual differences in episodic detail generation while remembering were reflected in future event sharing in daily conversation. Therefore, future studies will need to examine whether, with a greater sampling of shared future thoughts in daily conversation, stronger associations to older age are detectable.

In addition to investigating the frequency and detail make-up of autobiographical memory and future thought sharing, we also examined the degree to which a few laboratory-based outcomes on episodic detail generation were evident in the sharing of episodes in daily conversation. Similar to laboratory-based research (Palombo et al., 2015; Grilli et al., 2018a), we did not find a correlation between a list learning task and episodic detail generation while sharing autobiographical memories. Therefore, findings from both laboratory and naturalistic methods suggest that traditional verbal learning and memory tests may not provide much insight into the nature of real-world episodic specificity. More broadly, these results highlight the clinical importance of assessing episodic specificity of autobiographical memory sharing.

There were also two notable differences between laboratory-based work and our study of naturalistic autobiographical memory sharing. In our study, working memory was not associated with episodic specificity, a correlation that laboratory research has found in older adults. It is possible that our naturalistic sampling of autobiographical episodic memory sharing was not related to working memory because the memories captured were often shorter than what is reported in laboratory studies. We noticed that in their natural conversations, individuals often completed their description of a memory in less than the 30-s window of the EAR recording. Thus, it may be that working memory, perhaps in particular feature binding (i.e., episodic buffer; Baddeley, 2000; Piolino et al., 2010), is less important for the telling of shorter memories, and instead crucial for integrating and maintaining content over extended sharing. Another possibility is that our ability to capture a relationship was obscured given that only memory fragments were often captured. Future research using the EAR for this purpose could increase the recording time to better capture verbalized autobiographical memory sharing and examine different aspects of working memory more fully.

In addition to working memory, in contrast to laboratory-based work, we did not reveal gender differences in autobiographical memory sharing, as men and women were equally specific in their daily lives while conversing with others about past episodes. Although the current study did not directly address an underlying mechanism for the discrepancy between the findings of our naturalistic assessment and those of laboratory-bases studies, one possibility is that social and contextual interactions may modulate episodic specificity. In other words, men and women may express various degrees of autobiographical memory specificity depending on context (e.g., other individuals involved in the conversation; Aukett et al., 1988; Grysman and Hudson, 2013) and on the purpose that sharing serves (e.g., social bonding, teaching; Bluck, 2003). These factors may be critical to whether gender differences emerge. An alternative, and not mutually exclusive, explanation could be related to differences in available cues provided in naturalistic and laboratory environments. There is evidence that men may be more sensitive to visual/spatial cuing than women such that particular brain regions associated with greater detail, reliving, and richness showed greater activation to visuospatial compared to verbal cues in an autobiographical memory task (St. Jacques et al., 2011). These results might mean that the level of episodic specificity in men and women depends on the environmental cues of the experiment (i.e., visual/spatial cues in naturalistic studies and verbal cues in laboratory studies). Finally, many of the studies examining gender differences in autobiographical memory specificity were conducted in younger individuals (Wang et al., 2011; Fuentes and Desrocher, 2013; Wang, 2013). These findings have been extended to older individuals (Pillemer et al., 2003), but more research should be done to fully investigate possible gender differences in the specificity of socially shared personal episodes in older individuals.

The present study has a few main limitations that are worth considering. First, although episodic specificity differences have been found in laboratory-based studies of older adult cohorts (Piolino et al., 2002; De Beni et al., 2013), more often, an older adult group is compared to a young adult group. Thus, it may be that older adults, as a cohort, tend to generate more semantic memories in everyday life and provide more semantic details than young individuals, as would be predicted by executive coupling (Piolino et al., 2010; Turner and Spreng, 2015; Spreng et al., 2018) and narrative style theoretical models (James et al., 1998; Trunk and Abrams, 2009; Gaesser et al., 2011), but there is a relative decline in these features across older adulthood. A future study could address this possibility by evaluating autobiographical memory sharing across the adult age spectrum. A young vs. older adult comparison could further evaluate the degree to which other laboratory-based findings related to aging and autobiographical memory translate to real-world remembering in daily conversations, such as a relationship to working memory. Second, as is the case for studies of real-world behavior, our naturalistic recording approach inherently provides decreased experimental control over study conditions when compared to laboratory-based studies. Future research will need to examine whether real-world contextual features, such as with whom and where one is speaking, influence the relationship between older age and autobiographical memory. Such social and environmental differences could have contributed to the divergence between laboratory and real-world autobiographical memory assessment reported in the present study. In some respects, variability in environmental and social features can be an advantage, revealing contexts that prominently affect performance among older adults. We suggest that by pairing both in- and out-of-laboratory designs, one could strike a balance between the generalizability that naturalistic observation affords and the high level of experimental control of the laboratory. A third limitation is that the EAR can only capture verbalized memories and future thoughts, leaving in the dark memories and simulations that are part of internal cognition. This may be particularly noteworthy for the naturalistic assessment of future thinking, given that we captured fewer instances of sharing future thoughts (thus replicating Demiray et al., 2018). Other everyday assessment methods, such as diary studies or other thought sampling designs of self-reported vividness and content of future thoughts, may be better suited for capturing and assessing the quality of future thoughts. Fourth, with longer EAR snippets, we would be in a better position to examine the dynamic relationship between episodic and semantic detail use (Devitt et al., 2017). Relatedly, collecting EAR data over more than 4 days would likely boost the frequency of captured future thought-sharing in daily conversation, which may improve our ability to reliably evaluate the relationships between real-world autobiographical future thinking and older age. More days of EAR data collection would also presumably boost the frequency of autobiographical memories captured and variety of environmental contexts, which would allow for further investigation of how contextual features of daily conversation (e.g., with whom or where one is engaged in a conversation) relate to autobiographical memory sharing. Finally, while the selection of the two standard cognitive tests used in the present study was based on data from prior studies (Addis et al., 2008; Grilli et al., 2018a), not all participants received them and there was only one test per domain. A future study could administer a larger battery to more participants that includes a greater range of assessments for each cognitive construct.

Despite these limitations, the present study sheds new light on how older individuals share autobiographical memories in real-world contexts, and in the process, lays the foundation for future research using new technologies to further investigate a host of clinical and functional implications of memory sharing. On the clinical side, reduced episodic specificity has been documented in a range of neurologic conditions (e.g., dementia and amnesia, Irish et al., 2011, 2012; Palombo et al., 2018) and psychiatric conditions (e.g., schizophrenia and depression; Potheegadoo et al., 2014; Söderlund et al., 2014; MacDougall et al., 2015). Both in-laboratory and unobtrusive naturalistic acquisition of autobiographical memory could be useful clinical tools for tracking changes in episodic specificity in these populations. For example, evaluating baseline episodic specificity in-clinic or in-laboratory could indicate a need for follow-up assessments of specificity using naturalistic methods such as the EAR. Furthermore, including real-world observation of autobiographical memory could provide increased coherence in clinicians’ understanding of subjective patient complaints with observed deficits. Given that our standard working memory and verbal episodic memory tests were not significantly related to age, real-world memory assessment may be more sensitive to age-related cognitive changes than many laboratory-based cognitive tests, which tend to be socially decontextualized. However, we acknowledge that in future work, other standard cognitive tests will need to be compared to naturalistic assessment. On the functional side, autobiographical memory has been linked to performance on problem-solving tasks (Vandermorris et al., 2013; Madore and Schacter, 2014; McFarland et al., 2017), creative thinking (Madore et al., 2015), and emotion regulation (Jing et al., 2016). Therefore, naturalistic assessment of daily conversation using new technologies such as the EAR has the potential to be another important method for understanding the degree to which autobiographical memories are being adaptively applied to a variety of cognitive processes that are critical for wellbeing (Seifert et al., 2018).

In sum, in the present study, we used a new technology that has been recently applied to study memory sharing in natural social contexts (Demiray et al., 2018, 2019; Brianza and Demiray, 2019) and found that cross-sectionally, older individuals in the sample demonstrated lower frequencies of autobiographical memory sharing in everyday conversations, a relationship that affected both episodic and semantic memories. For episodic memories, older age was also linked to a reduction in the level of detail at which events were described. In terms of future autobiographical thought sharing, age was neither related to sharing of future episodic thoughts nor sharing of all details in future episodic thoughts. Interestingly, age was negatively correlated with future semantic thought sharing. Additional analyses revealed a commonality with findings from laboratory research (i.e., a lack of a relationship between specificity and a cognitive test of episodic memory) and differences (i.e., lack of a relationship between episodic detail in memories and future thoughts, lack of a relationship between episodic specificity and working memory, and gender differences). Overall, our findings align with recent research showing that the EAR and similar new technologies for unobtrusively capturing cognition “in the wild” can complement laboratory-based approaches and provide new insights into the actual use of autobiographical memory in everyday life.

## Data Availability Statement

The dataset analyzed for this study and the corresponding data analysis code can be found here: https://osf.io/f3euv/.

## Ethics Statement

The studies involving human participants were reviewed and approved by Institutional Review Board University of Arizona. The participants provided their written informed consent to participate in this study.

## Author Contributions

Contributor Role Taxonomy (CRediT) taxonomy. AW: methodology, formal analysis, data curation, writing—original draft, writing—reviewing and editing, and visualization. MM: conceptualization, methodology, writing—reviewing and editing, and supervision. JA-H: conceptualization, writing—reviewing and editing. AP: investigation, data curation, project administration, and funding acquisition. SM: investigation, data curation, and project administration. EG: investigation, resources, writing—reviewing and editing, and supervision. MG: conceptualization, methodology, resources, supervision of formal analysis, writing—original draft, writing—review and editing, and supervision.

## Conflict of Interest

The authors declare that the research was conducted in the absence of any commercial or financial relationships that could be construed as a potential conflict of interest. The handling Editor declared a past co-authorship with one of the author MM.

## Footnotes

^1^Education was not significantly related to age, gender, or any of the variables reported in the “Results” section.

^2^Mini-Mental Status Examination scores for two participants were not available.

^3^Because 23 participants only had long delay cued recall scores from the first edition of the CVLT, we ran the correlation with EAR episodic specificity separately for those with first edition CVLT scores and those with second edition CVLT scores. In both cases, we obtained the same results (i.e., no correlation between episodic memory and past episodic specificity).

## References

[B1] Addis D. R. (2018). Are episodic memories special? On the sameness of remembered and imagined event simulation. J. R. Soc. N. Z. 48, 64–88. 10.1080/03036758.2018.1439071

[B2] Addis D. R. Wong A. T. Schacter D. L. (2008). Age-related changes in the episodic simulation of future events. Psychol. Sci. 19, 33–41. 10.1111/j.1467-9280.2008.02043.x 18181789

[B3] Alea N. Bluck S. (2003). Why are you telling me that? A conceptual model of the social function of autobiographical memory. Memory 11, 165–178. 10.1080/741938207 12820829

[B4] Alea N. Bluck S. (2007). I’ll keep you in mind: the intimacy function of autobiographical memory. Appl. Cogn. Psychol. 21, 1091–1111. 10.1002/acp.1316

[B5] Andreano J. M. Cahill L. (2009). Sex influences on the neurobiology of learning and memory. Learn. Mem. 16, 248–266. 10.1101/lm.918309 19318467

[B6] Andrews-Hanna J. R. Grilli M. D. Irish M. (2019). “A review and reappraisal of the default network in normal aging and dementia,” in Oxford Research Encyclopedia of Psychology, eds Knight R. T. Neupert S. D. Anderson N. D. Wahl H. W. Pachana N. A. (Oxford: Oxford University Press).

[B7] Andrews-Hanna J. R. Smallwood J. Spreng R. N. (2014). The default network and self-generated thought: component processes, dynamic control, and clinical relevance. Ann. N Y Acad. Sci. 1316, 29–52. 10.1111/nyas.12360 24502540 PMC4039623

[B8] Andrews-Hanna J. R. Snyder A. Z. Vincent J. L. Lustig C. Head D. Raichle M. E.. (2007). Disruption of large-scale brain systems in advanced aging. Neuron 56, 924–935. 10.1016/j.neuron.2007.10.038 18054866 PMC2709284

[B9] Auguie B. (2017). gridExtra: Miscellaneous Functions for “Grid” Graphics. R package version 2.3. Available online at: https://CRAN.R-project.org/package=gridExtra. Accessed May 13, 2020.

[B10] Aukett R. Ritchie J. Mill K. (1988). Gender differences in friendship patterns. Sex Roles 19, 57–66. 10.1007/BF00292464

[B11] Baddeley A. (2000). The episodic buffer: a new component of working memory? Trends Cogn. Sci. 4, 417–423. 10.1016/s1364-6613(00)01538-2 11058819

[B12] Barnabe A. Whitehead V. Pilon R. Arsenault-Lapierre G. Chertkow H. (2012). Autobiographical memory in mild cognitive impairment and Alzheimer’s disease: a comparison between the Levine and Kopelman interview methodologies. Hippocampus 22, 1809–1825. 10.1002/hipo.22015 22488637

[B13] Bastin C. Feyers D. Jedidi H. Bahri M. A. Degueldre C. Lemaire C.. (2013). Episodic autobiographical memory in amnestic mild cognitive impairment: what are the neural correlates? Hum. Brain Mapp. 34, 1811–1825. 10.1002/hbm.22032 22422512 PMC6869917

[B14] Berntsen D. Hall N. M. (2004). The episodic nature of involuntary autobiographical memories. Mem. Cognit. 32, 789–803. 10.3758/bf03195869 15552356

[B15] Bluck S. (2003). Autobiographical memory: exploring its functions in everyday life. Memory 11, 113–123. 10.1080/741938206 12820825

[B17] Bluck S. Alea N. (2002). “Exploring the functions of autobiographical memory: why do I remember the autumn?” in Critical Advances of Reminiscence Work, eds Webster J. D. Haight B. K. (New York, NY: Springer Publishing Company), 61–75.

[B16] Bluck S. Alea N. Habermas T. Rubin D. C. (2005). A Tale of three functions: the self-reported uses of autobiographical memory. Soc. Cogn. 23, 91–117. 10.1521/soco.23.1.91.59198

[B18] Brianza E. Demiray B. (2019). Future time perspective and real-life utterances about the future in young and older adults. GeroPsych 32, 161–173. 10.1024/1662-9647/a000216

[B19] Conway M. A. Pleydell-Pearce C. W. (2000). The construction of autobiographical memories in the self-memory system. Psychol. Rev. 107, 261–288. 10.1037/0033-295x.107.2.261 10789197

[B20] D’Argembeau A. Renaud O. Van der Linden M. (2011). Frequency, characteristics, and functions of future-oriented thoughts in daily life. Appl. Cogn. Psychol. 25, 96–103. 10.1002/acp.1647

[B21] Davis P. J. (1999). Gender differences in autobiographical memory for childhood emotional experiences. J. Pers. Soc. Psychol. 76, 498–510. 10.1037/0022-3514.76.3.498 10101879

[B22] De Beni R. Borella E. Carretti B. Zavagnin M. Lazzarini L. Milojevi G. (2013). Remembering the past and imagining the future: age-related differences between young, young-old and old-old. Aging Clin. Exp. Res. 25, 89–97. 10.1007/s40520-013-0003-3 23740638

[B23] De Brigard F. Giovanello K. S. Stewart G. W. Lockrow A. W. O’Brien M. M. Spreng R. N. (2016). Characterizing the subjective experience of episodic past, future, and counterfactual thinking in healthy younger and older adults. Q. J. Exp. Psychol. 69, 2358–2375. 10.1080/17470218.2015.1115529 27028484

[B24] Delis D. C. Kramer J. H. Kaplan E. Ober B. A. (2000). California Verbal Learning Test, Second Edition (CVLT-II). San Antonio, TX: Psychological Corporation.

[B25] Delis D. C. Kramer J. Kaplan E. Ober B. A. Fridlund A. (1987). The California Verbal Learning Test. New York, NY: Psychological Corporation.

[B26] Demiray B. Mehl M. R. Martin M. (2018). Conversational time travel: evidence of a retrospective bias in real life conversations. Front. Psychol. 9:2160. 10.3389/fpsyg.2018.02160 30483183 PMC6243041

[B27] Demiray B. Mischler M. Martin M. (2019). Reminiscence in everyday conversations: a naturalistic observation study of older adults. J. Gerontol. B Psychol. Sci. Soc. Sci. 74, 745–755. 10.1093/geronb/gbx141 29190392

[B28] Devitt A. L. Addis D. R. Schacter D. L. (2017). Episodic and semantic content of memory and imagination: a multilevel analysis. Mem. Cognit. 45, 1078–1094. 10.3758/s13421-017-0716-1 28547677 PMC5702280

[B29] Diedenhofen B. Musch J. (2015). Cocor: a comprehensive solution for the statistical comparison of correlations. PLoS One 10:e0121945. 10.1371/journal.pone.0121945 25835001 PMC4383486

[B30] Fjell A. M. Westlye L. T. Amlien I. Espeseth T. Reinvang I. Ras N.. (2009). High consistency of regional cortical thinning in aging across multiple samples. Cereb. Cortex 19, 2001–2012. 10.1093/cercor/bhn232 19150922 PMC2733683

[B31] Ford J. H. Rubin D. C. Giovanello K. S. (2014). Effects of task instruction on autobiographical memory specificity in young and older adults. Memory 22, 722–736. 10.1080/09658211.2013.820325 23915176 PMC3916949

[B32] Fox J. Weisberg S. (2019). An R Companion to Applied Regression, Third Edition. Thousand Oaks, CA: Sage.

[B33] Fuentes A. Desrocher M. (2013). The effects of gender on the retrieval of episodic and semantic components of autobiographical memory. Memory 21, 619–632. 10.1080/09658211.2012.744423 23240928

[B34] Gaesser B. (2013). Constructing memory, imagination, and empathy: a cognitive neuroscience perspective. Front. Psychol. 3:576. 10.3389/fpsyg.2012.00576 23440064 PMC3579581

[B35] Gaesser B. Schacter D. L. (2014). Episodic simulation and episodic memory can increase intentions to help others. Proc. Natl. Acad. Sci. U S A 111, 4415–4420. 10.1073/pnas.1402461111 24616532 PMC3970486

[B36] Gaesser B. Sacchetti D. C. Addis D. R. Schacter D. L. (2011). Characterizing age-related changes in remembering the past and imagining the future. Psychol. Aging 26, 80–84. 10.1037/a0021054 21058863 PMC3062729

[B38] Grilli M. D. Wank A. A. Bercel J. J. Ryan L. (2018a). Evidence for reduced autobiographical memory episodic specificity in cognitively normal middle-aged and older individuals at increased risk for Alzheimer’s disease dementia. J. Int. Neuropsychol. Soc. 24, 1073–1083. 10.1017/s1355617718000577 30136918 PMC6237636

[B37] Grilli M. D. Wank A. A. Verfaellie M. (2018b). The life stories of adults with amnesia: insights into the contribution of the medial temporal lobes to the organization of autobiographical memory. Neuropsychologia 110, 84–91. 10.1016/j.neuropsychologia.2017.03.013 28286259 PMC5592132

[B39] Grysman A. Hudson J. A. (2013). Gender differences in autobiographical memory: developmental and methodological considerations. Dev. Rev. 33, 239–272. 10.1016/j.dr.2013.07.004

[B40] Hill P. F. Emery L. J. (2013). Episodic future thought: contributions from working memory. Conscious. Cogn. 22, 677–683. 10.1016/j.concog.2013.04.002 23681207

[B41] Hodgetts C. J. Postans M. Warne N. Varnava A. Lawrence A. D. Graham K. S. (2017). Distinct contributions of the fornix and inferior longitudinal fasciculus to episodic and semantic autobiographical memory. Cortex 94, 1–14. 10.1016/j.cortex.2017.05.010 28710907 PMC5576916

[B42] Irish M. Addis D. R. Hodges J. R. Piguet O. (2012). Considering the role of semantic memory in episodic future thinking: evidence from semantic dementia. Brain 135, 2178–2191. 10.1093/brain/aws119 22614246

[B43] Irish M. Hornberger M. Lah S. Miller L. Pengas G. Nestor P. J.. (2011). Profiles of recent autobiographical memory retrieval in semantic dementia, behavioural-variant frontotemporal dementia and Alzheimer’s disease. Neuropsychologia 49, 2694–2702. 10.1016/j.neuropsychologia.2011.05.017 21658396

[B44] James L. E. Burke D. M. Austin A. Hulme E. (1998). Production and perception of “verbosity” in younger and older adults. Psychol. Aging 13, 355–367. 10.1037/0882-7974.13.3.355 9793112

[B45] Jing H. G. Madore K. P. Schacter D. L. (2016). Worrying about the future: an episodic specificity induction impacts problem solving, reappraisal and well-being. J. Exp. Psychol. Gen. 145, 402–418. 10.1037/xge0000142 26820166 PMC4792686

[B46] Kim S. (2015). ppcor: Partial and Semi-Partial (part) Correlation. R package version 1.1. Available online at: https://CRAN.R-project.org/package=ppcor. Accessed May 13, 2020.

[B47] Lambon Ralph M. A. Ehsan S. Baker G. A. Rogers T. T. (2012). Semantic memory is impaired in patients with unilateral anterior temporal lobe resection for temporal lobe epilepsy. Brain 135, 242–258. 10.1093/brain/awr325 22287382 PMC3267985

[B48] Levine B. Svoboda E. Hay J. F. Winocur G. Moscovitch M. (2002). Aging and autobiographical memory: dissociating episodic from semantic retrieval. Psychol. Aging 17, 677–689. 10.1037/0882-7974.17.4.677 12507363

[B49] MacDonald S. Uesiliana K. Hayne H. (2000). Cross-cultural and gender differences in childhood amnesia. Memory 8, 365–376. 10.1080/09658210050156822 11145068

[B50] MacDougall A. G. McKinnon M. C. Herdman K. A. King M. J. Kiang M. (2015). The relationship between insight and autobiographical memory for emotional events in schizophrenia. Psychiatry Res. 226, 392–395. 10.1016/j.psychres.2014.12.058 25623015

[B51] Madore K. P. Addis D. R. Schacter D. L. (2015). Creativity and memory: effects of an episodic-specificity induction on divergent thinking. Psychol. Sci. 26, 1461–1468. 10.1177/0956797615591863 26205963 PMC4567456

[B53] Madore K. P. Gaesser B. Schacter D. L. (2014). Constructive episodic simulation: dissociable effects of a specificity induction on remembering, imagining, and describing in young and older adults. J. Exp. Psychol. Learn. Mem. Cogn. 40, 609–622. 10.1037/a0034885 24188466 PMC4006318

[B52] Madore K. P. Schacter D. L. (2014). An episodic specificity induction enhances means-end problem solving in young and older adults. Psychol. Aging 29, 913–924. 10.1037/a0038209 25365688 PMC4268420

[B54] McFarland C. P. Primosch M. Maxon C. M. Stewart B. T. (2017). Enhancing memory and imagination improves problem solving among individuals with depression. Mem. Cognit. 45, 932–939. 10.3758/s13421-017-0706-3 28405957

[B55] Mehl M. R. (2017). The electronically activated recorder (EAR): a method for the naturalistic observation of daily social behavior. Curr. Dir. Psychol. Sci. 26, 184–190. 10.1177/0963721416680611 28529411 PMC5434514

[B56] Mehl M. R. Holleran S. E. (2007). An empirical analysis of the obtrusiveness of and participants’ compliance with the electronically activated recorder (EAR). Eur. J. Psychol. Assess. 23, 248–257. 10.1027/1015-5759.23.4.248

[B57] Mehl M. R. Pennebaker J. W. Crow D. M. Dabbs J. Price J. H. (2001). The electronically activated recorder (EAR): a device for sampling naturalistic daily activities and conversations. Behav. Res. Methods 33, 517–523. 10.3758/bf03195410 11816455

[B58] Memel M. Wank A. A. Ryan L. Grilli M. D. (2020). The relationship between episodic detail generation and anterotemporal, posteromedial, and hippocampal white matter tracts. Cortex 123, 124–140. 10.1016/j.cortex.2019.10.010 31783222 PMC6984983

[B59] Moseley S. (2018). Cognitive and Psychosocial Associations of Hearing Loss in Older Adults. Tucson, AZ: University of Arizona. Dissertation.

[B60] Murphy K. J. Troyer A. K. Levine B. Moscovitch M. (2008). Episodic, but not semantic, autobiographical memory is reduced in amnestic mild cognitive impairment. Neuropsychologia 46, 3116–3123. 10.1016/j.neuropsychologia.2008.07.004 18675285 PMC2629588

[B61] Niedźwieńska A. (2003). Gender differences in vivid memories. Sex Roles 49, 321–331. 10.1023/A:1025156019547

[B62] Palombo D. J. Alain C. Söderlund H. Khuu W. Levine B. (2015). Severely deficient autobiographical memory (SDAM) in healthy adults: a new mnemonic syndrome. Neuropsychologia 72, 105–118. 10.1016/j.neuropsychologia.2015.04.012 25892594

[B63] Palombo D. J. Sheldon S. Levine B. (2018). Individual differences in autobiographical memory. Trends Cogn. Sci. 22, 583–597. 10.1016/j.tics.2018.04.007 29807853

[B64] Pasupathi M. Lucas S. Coombs A. (2002). Conversational functions of autobiographical remembering: long-married couples talk about conflicts and pleasant topics. Discourse Process. 34, 163–192. 10.1207/s15326950dp3402_3

[B65] Pillemer D. B. (1992). “Remembering personal circumstances: a functional analysis,” in Affect and Accuracy in Recall: Studies of “Flashbulb” Memories, eds Winograd E. Neisser U. (New York, NY: Cambridge University Press), 236–264.

[B66] Pillemer D. B. Wink P. DiDonato T. E. Sanborn R. L. (2003). Gender differences in autobiographical memory styles of older adults. Memory 11, 525–532. 10.1080/09658210244000117 14982120

[B67] Piolino P. Coste C. Martinelli P. Macé L. Quinette P. Guillery-Girard B.. (2010). Reduced specificity of autobiographical memory and aging: do the executive and feature binding functions of working memory have a role? Neuropsychologia 48, 429–440. 10.1016/j.neuropsychologia.2009.09.035 19804792

[B68] Piolino P. Desgranges B. Benali K. Eustache F. (2002). Episodic and semantic remote autobiographical memory in ageing. Memory 10, 239–257. 10.1080/09658210143000353 12097209

[B69] Piolino P. Desgranges B. Clarys D. Guillery-Girard B. Taconnat L. Isingrini M.. (2006). Autobiographical memory, autonoetic consciousness, and self-perspective in aging. Psychol. Aging 21, 510–525. 10.1037/0882-7974.21.3.510 16953713

[B70] Polsinelli A. (2017). Cognitive and Emotional Associations of Mindfulness in Older Adults. Tucson, AZ: University of Arizona. Dissertation.

[B71] Polsinelli A. J. Moseley S. A. Grilli M. D. Glisky E. L. Mehl M. R. (2020). Natural, everyday language use provides a window into the integrity of older adults’ executive functioning. J. Gerontol. B Psychol. [Epub ahead of print]. 10.1093/geronb/gbaa055 32310293

[B72] Potheegadoo J. Cordier A. Berna F. Danion J. M. (2014). Effectiveness of a specific cueing method for improving autobiographical memory recall in patients with schizophrenia. Schizophr. Res. 152, 229–234. 10.1016/j.schres.2013.10.046 24268933

[B73] R Core Team (2019). R: A Language and Environment for Statistical Computing. R Foundation for Statistical Computing. Vienna: Austria Available online at: https://www.R-project.org/. Accessed May 13, 2020.

[B74] Robbins M. L. (2017). Practical suggestions for legal and ethical concerns with social environment sampling methods. Soc. Psychol. Pers. Sci. 8, 573–580. 10.1177/1948550617699253

[B75] Ros L. Latorre J. M. Serrano J. P. (2009). Working memory capacity and overgeneral autobiographical memory in young and older adults. Neuropsychol. Dev. Cogn. B Aging Neuropsychol. Cogn. 17, 89–107. 10.1080/13825580903042650 19626477

[B76] Ros L. Latorre J. M. Serrano J. P. Ricarte J. J. (2017). Overgeneral autobiographical memory in healthy young and older adults: differential age effects on components of the Capture and Rumination, Functional Avoidance and Impaired Executive Control (CaRFAX) model. Psychol. Aging 32, 447–459. 10.1037/pag0000175 28594191

[B77] Schacter D. L. Addis D. R. (2007). The cognitive neuroscience of constructive memory: remembering the past and imagining the future. Philos. T. R. Soc. B. 362, 773–786. 10.1098/rstb.2007.2087 17395575 PMC2429996

[B78] Schlagman S. Kvavilashvili L. (2008). Involuntary autobiographical memories in and outside the laboratory: how different are they from voluntary autobiographical memories? Mem. Cognit. 36, 920–932. 10.3758/mc.36.5.920 18630199

[B79] Seifert A. Hofer M. Allemand M. (2018). Mobile data collection: smart, but not (yet) smart enough. Front. Neurosci. 12:971. 10.3389/fnins.2018.00971 30618590 PMC6305304

[B80] Söderlund H. Moscovitch M. Kumar N. Daskalakis Z. J. Flint A. Herrmann N.. (2014). Autobiographical episodic memory in major depressive disorder. J. Abnorm. Psychol. 123, 51–60. 10.1037/a0035610 24661159

[B81] Spreng R. N. Lockrow A. W. DuPre E. Setton R. Spreng K. A. P. Turner G. R. (2018). Semanticized autobiographical memory and the default-executive coupling hypothesis of aging. Neuropsychologia 110, 37–43. 10.1016/j.neuropsychologia.2017.06.009 28624521

[B83] St. Jacques P. L. Conway M. A. Cabeza R. (2011). Gender differences in autobiographical memory for everyday events: retrieval elicited by SenseCam images versus verbal cues. Memory 19, 723–732. 10.1080/09658211.2010.516266 20981611 PMC3190064

[B82] St. Jacques P. L. Levine B. (2007). Ageing and autobiographical memory for emotional and neutral events. Memory 15, 129–144. 10.1080/09658210601119762 17534107 PMC1995658

[B84] Steiger J. H. (1980). Tests for comparing elements of a correlation matrix. Psychol. Bull. 87, 245–251. 10.1037/0033-2909.87.2.245

[B85] Trunk D. L. Abrams L. (2009). Young and older adults’ communicative goals influence off-topic speech in autobiographical narratives? Psychol. Aging 24, 324–337. 10.1037/a0015259 19485651

[B86] Turner G. R. Spreng R. N. (2015). Prefrontal engagement and reduced default network suppression co-occur and are dynamically coupled in older adults: the default-executive coupling hypothesis of aging. J. Cogn. Neurosci. 27, 2462–2476. 10.1162/jocn_a_00869 26351864

[B87] Vandermorris S. Sheldon S. Winocur G. Moscovitch M. (2013). Differential contributions of executive and episodic memory functions to problem solving in younger and older adults. J. Int. Neuropsychol. Soc. 19, 1087–1096. 10.1017/S1355617713000982 24044692

[B88] Verfaellie M. Bousquet K. Keane M. M. (2014). Medial temporal and neocortical contributions to remote memory for semantic narratives: evidence from amnesia. Neuropsychologia 61, 105–112. 10.1016/j.neuropsychologia.2014.06.018 24953960 PMC4122606

[B89] Wang Q. (2013). Gender and emotion in everyday event memory. Memory 21, 503–511. 10.1080/09658211.2012.743568 23190136

[B90] Wang Q. Hou Y. Tang H. Wiprovnick A. (2011). Travelling backwards and forwards in time: culture and gender in the episodic specificity of past and future events. Memory 19, 103–109. 10.1080/09658211.2010.537279 21240752

[B91] Waters T. E. A. Bauer P. J. Fivush R. (2014). Autobiographical memory functions served by multiple event types. Appl. Cogn. Psychol. 28, 185–195. 10.1002/acp.2976

[B92] Wechsler D. (1997). Wechsler Adult Intelligence Scale—Third Edition. San Antonio, TX: Psychological Corporation.

[B93] Wickham H. (2007). Reshaping data with the reshape package. J. Stat. Softw. 21, 1–20. 10.18637/jss.v021.i12

[B94] Wickham H. (2016). ggplot2: Elegant Graphics for Data Analysis. New York, NY: Springer-Verlag.

[B95] Williams J. M. Broadbent K. (1986). Autobiographical memory in suicide attempters. J. Abnorm. Psychol. 95, 144–149. 10.1037/0021-843x.95.2.144 3711438

